# Maternal Healthcare Service Utilization in Bangladesh: A Cross‐Sectional Study of Determinants and Temporal Trends Using BDHS 2011–2022

**DOI:** 10.1002/hsr2.72171

**Published:** 2026-03-26

**Authors:** Kamrul Hassan Prantik, Anika Tabassum Nabi, Golam Morshed Suhel, Mahmud Afroz, Mohammad Nayeem Hasan

**Affiliations:** ^1^ Department of Statistics Shahjalal University of Science and Technology Sylhet Bangladesh; ^2^ Department of Sociology University at Albany, State University of New York Albany New York USA; ^3^ Department of Geosciences University of Arkansas Fayetteville Arkansas USA

**Keywords:** antenatal care, facility‐based delivery, skilled birth attendance, socio‐demographic determinants, temporal trends

## Abstract

**Background:**

In recent decades, Bangladesh has improved maternal health outcomes significantly. However, disparities in access to maternal healthcare facilities over time remain, particularly in a densely populated country like Bangladesh. This research seeks to highlight the growing use of maternal health facilities from 2011 to 2022 and to reveal key determinants influencing utilization.

**Method:**

The analysis used dataset extracted from the 2011, 2014, 2017–2018, and 2022 rounds of Bangladesh Demographic Health Survey (BDHS). Temporal trends in maternal health services across survey years are depicted by descriptive statistics. Overall utilization was examined using pooled data. Multivariable logistic regression identified the associated factors influencing the usage of maternal healthcare services.

**Results:**

The study revealed an upward trend in the use of maternal health facilities throughout the survey years. In 2022, women were 1.57 times more likely to have four Antenatal care visits, 5.36 (95% CI: 4.34–6.63) times more likely to have facility‐based delivery, and 5.61 (95% CI: 4.63–6.79) times more likely to receive skilled birth attendance compared to 2011. Higher utilization was associated with urban residence with odds of 1.35 (95% CI: 1.16–1.58), 1.28 (95% CI: 1.09–1.51), and 1.34 (95% CI: 1.16–1.55) for ANC, FBA, and SBA, respectively. Higher maternal education showed odds of 4.59 (95% CI: 3.27–6.43), 4.44 (95% CI: 3.14–6.29), and 6.07 (95% CI:4.52–8.16), and richest households utilized maternal health care services 2.59 (95% CI: 1.99–3.38), 4.64 (95% CI: 3.53–6.10), and 4.34 (95% CI: 3.40–5.43) times more for ANC, FBA, and SBA, respectively.

**Conclusion:**

This study demonstrates significant improvements in maternal healthcare engagement in Bangladesh over the past decade. Exposure to media and longer birth intervals were associated with higher utilization. Regional, religious, and household‐level disparities were also evident, whereas factors such as employment and larger family size were linked to reduced service use. However, disparities remain across regions, socioeconomic groups, and household characteristics, underscoring the need for targeted interventions.

**Abbreviations**
ANC: antenatal care
BDHS: Bangladesh Demographic Health Survey
FBD: facility based delivery
MHC: maternal healthcare
SBA: skilled birth attendant

## 1 Introduction

Maternal healthcare facilities, including antenatal care visits, professional birth assistance, and postnatal checkups play an important role in reduction of maternal and newborn mortality, as well as in avoiding other related problems [1]. Studies show that a 1% increase in maternal healthcare services utilization can reduce maternal death by 0.35 per 100,000 live births [2]. Access to maternal healthcare facilities is still a challenge for millions of women, particularly in low‐ and middle‐income nations [3]. Notable disparities in the usage of antenatal care and skilled birth attendance were found across continents, with Asia and Africa exhibiting the most pronounced differences [4]. Failure to receive timely and adequate maternal healthcare services significantly escalates the risk of negative outcomes, including maternal complications, insufficient gestational weight gain, and negative health behaviors such as prenatal smoking, all of which can harm both maternal and neonatal health [5, 6]. Approximately 810 women globally succumb to preventable complications related to pregnancy and childbirth, with 94% of these maternal deaths occurring in low‐income or resource‐constrained environments [7].

Geographic location, educational attainment, and socioeconomic status can significantly contribute to the inaccessibility of maternal healthcare facilities [8, 9]. A study showed that women who had secondary and higher levels of education had 25% higher likelihood of utilizing maternal healthcare services than women who had primary education or no education at all [10]. Maternal mortality underscores substantial disparities in access to fundamental healthcare, particularly between women from affluent socioeconomic backgrounds and those from underprivileged areas. Urban women had a greater likelihoods of utilizing antenatal care, institutional deliveries, and skilled birth assistance in comparison to women in rural regions [11, 12]. A further study indicates that household wealth status significantly influences inequality in access to adequate maternal healthcare services, accounting for 41.4%. This encompasses having a minimum of four antenatal care visits (39.7%), obtaining appropriate ANC services (50.7% and 44.0%), and giving birth in healthcare facilities (43.4%) [13].

Maternal healthcare in Bangladesh is still a critical public health issue, influenced by a complex interplay of socioeconomic, cultural, and structural variables [14]. A study showed that approximately three‐fourths of deliveries are conducted by traditional birth attendants (TBAs) at home [15]. In Bangladesh, several factors affect low usage of maternal health care services, such as inadequate communication, insufficient awareness of available services, financial constraints, limited decision‐making power, and not having a companion to attend healthcare visits [16]. Moreover, maternal healthcare decisions are strongly influenced by cultural beliefs and traditions. A study in Bangladesh's coastal areas found that cultural beliefs limit pregnant women's access to healthcare services more than other factors by 2.73 times. Additionally, greater distance from healthcare services was associated with a 1.22 times higher likelihood of restricted access compared to other factors [17]. Beyond that, many cultures prefer home births over hospital deliveries, with TBAs present. These practices raise the possibility of complications and maternal death because TBAs do not have the knowledge or instruments which needed to handle obstetric emergencies [18].

While previous studies have explored trends up to 2011, as well as the determinants and urban‐rural disparities in maternal healthcare service utilization using BDHS data [19, 20], this study provides an updated and more comprehensive analysis by incorporating trends and pooled data from the most recent BDHS rounds (2011–2022). In contrast to earlier research, this study includes an expanded set of explanatory variables that capture socio‐demographic and health system‐related factors influencing maternal healthcare and the distribution of key explanatory variables across each survey year was systematically examined, offering an understanding of temporal trends and contextual changes over time. This enhanced temporal scope and analytical depth contribute to a more comprehensive view of the factors influencing maternal health service utilization in Bangladesh which can aid policymakers in prioritizing resource distribution, customizing awareness initiatives, and enhancing access to services for marginalized communities. This study ultimately supports national initiatives to enhance maternal health systems and advance toward the attainment of Sustainable Development Goal 3.1 – decreasing the rate of maternal mortality [21].

## 2 Methodology

### 2.1 Data Source and Study Design

**FIGURE 1:**
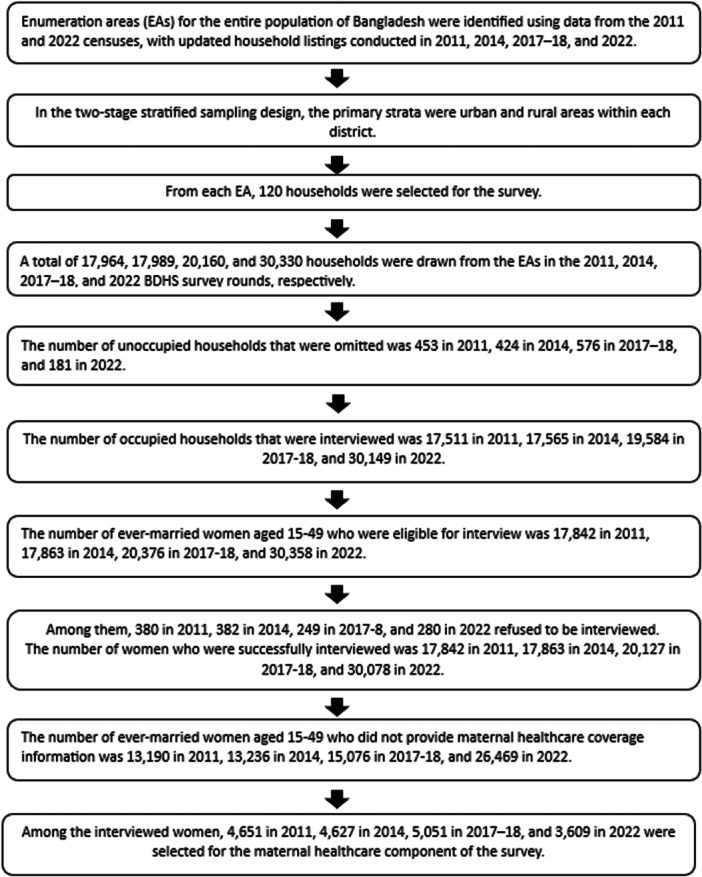
Overview of the study design across BDHS survey years 2011, 2014, 2017–2018, and 2022.

This study adhered to the STROBE guidelines to ensure comprehensive and accurate reporting of observational research (see Supporting File 1). The analysis utilized data from the most recent four survey years 2011, 2014, 2017–2018, and 2022 of Bangladesh Demographic Health Survey (BDHS). To assess trends and identify factors influencing access to maternal healthcare services–specifically antenatal care visits, place of delivery and skilled birth assistance–the data from these survey rounds were pooled for analysis. The study focused on women who are aged 15–49 and had a live birth in the 3 years preceding each survey. As shown in Figure 1, the number of observations included from the BDHS survey years 2011, 2014, 2017–2018, and 2022 were 4652, 4627, 5051, and 3609, respectively. After pooling the datasets from these survey years, the total number of observations was 17,939.

### 2.2 Outcome Variables

ANC: In our study antenatal care visits were categorized into two groups: women who had less than four ANC visits (“< 4”) and those who had at least four or more visits (“≥ 4”) [22, 23].

FBD: Facility‐based delivery refers to childbirth that takes place in a healthcare facility equipped with essential medical resources [24]. The respondents were categorized into two groups: whether a woman had given birth in “No healthcare facility” or “healthcare facility.”

SBA: Skilled birth assistance means the delivery of a child in the presence of qualified healthcare professionals [25]. In this study respondents were categorized whether they had skilled birth attendance “Yes” or “No.”

### 2.3 Independent Variables

A comprehensive review of existing literature was carried out to identify the key independent variables related with maternal health service utilization, which formed the basis of this study [22, 26]. This study incorporated a set of explanatory variables, including demographic, socioeconomic, and household‐level factors. Participants' residences were classified as either “urban” or “rural,” division was classified into “Dhaka,” “Barisal,” “Chittagong,” “Khulna,” “Mymensingh,” “Rajshahi,” “Rangpur,” and “Sylhet.” Religion was grouped into “Islam” and “Others.” Mother's age was categorized into “15–19,” “20–24,” and “25+” years. Mother's and Husband's education was classified as “No education,” “Primary,” “Secondary,” and “Higher.” The mother's current employment status was recorded as “Yes” or “No.” Husband's school type was categorized into “School” and “Madrasa.” Husband's occupation was classified as “Not working,” “Farming/Agriculture,” “Worker/Jobholder,” and “Others.” Marital duration was categorized into “0–4,” “5–9,” “10–14,” “15–19,” “20–24,” and “25+” years. Reproductive history included “Wanted pregnancy” with categories “Then,” “Later,” and “No more.” The length of the preceding birth interval was grouped into “< 24 months,” “24–59 months,” and “60+ months.” Birth order was categorized into “1,” “2,” and “3+.” Decision‐making authority for healthcare was divided into “Woman alone,” “Woman and partner,” “Partner alone,” and “Someone else.” Household head's gender (“Male” and “Female”) and age of household head was grouped into “15–24,” “25–29,” “30–34,” and “35+” years. The number of children was categorized into “Less than 2” and “More than 2,” and the number of household members was grouped as “Greater than 5” and “Less than or equal to 5.” The wealth index was kept into five quintiles: “Poorest,” “Poorer,” “Middle,” “Richer,” as BDHS report, and “Richest,” Media access was recorded as “Yes” or “No.” The drinking water's source and toilet facility was classified into “Improved” and “Unimproved.” These attributes were determined as per their proven relevance in maternal and child healthcare, as highlighted in both regional and global studies, and because they were available in the BDHS dataset [27, 28].

### 2.4 Statistical Analysis and Software

Initially, the distribution of demographic and socioeconomic characteristics information for each survey year was calculated. We merged data from all three surveys into a single dataset. During this process, Stata identified instances of a single primary sampling unit (PSU) within some strata. To address this, we applied the “singleton (scaled)” method, which adjusts for strata containing only one PSU‐common when combining datasets or due to missing or incomplete data. This approach was appropriate since each survey round uses distinct PSUs, and the scaled method ensures valid variance estimation across the combined dataset.

Thereafter, the trends in the access of maternal healthcare services were examined over time. Chi‐square test was conducted on the pooled dataset to find the association among the dependent and the independent variables. Variables showing a *p*‐value < 0.05 were considered to have significant association were chosen for additional examination. The study employed multivariable logistic regression models to generate 95% CI and adjusted odds ratios (AORs). The analysis was performed in Stata version 17 and R version 4.4.2. Stata's survey estimation procedures (the “svy” command) was used for Sampling weights for the BDHS dataset to obtain nationally representative estimates, accounting for sample clustering [29].

### 2.5 Model Evaluation

Multicollinearity was examined utilizing the Variance Inflation Factor (VIF). Variables with a value greater than 4 were excluded from the model. To evaluate model discrimination, the Receiver Operating Characteristic (ROC) curve was utilized while Hosmer–Lemeshow goodness of fit test was used to evaluate model calibration. Additionally, for model comparison and selection, the Bayesian Information Criterion (BIC) and the Akaike Information Criterion (AIC) were employed. An AUROC greater than 0.50 signifies the model's capability to discriminate between outcome categories, and a low *p*‐value indicates the statistical significance of this discriminative power [30, 31]. A *p*‐value of more than 0.05 in the Hosmer–Lemeshow test implies that there is no significant difference between the expected and observed results, indicating a good model fit [32, 33]. AIC and BIC with lower values indicated a better balance between model complexity and fit. Classification accuracy was evaluated to assess the predictive ability of the model.

## 3 Results

Table 1 demonstrates the socio‐demographic information of the respondents from BDHS surveys from 2011 to 2022. The proportion of urban mothers increased slightly from 22.8% in 2011 to 26.9% in 2022, while the majority remained rural (73.1% in 2022). Dhaka consistently had the highest share of respondents, accounting for 25.0% in 2022. Over time, mothers' education levels improved significantly: the proportion with no education declined from 17.4% in 2011 to 4.9% in 2022, while those with secondary education rose from 45.0% to 54.2%. Most mothers were aged 20–24 (33.7% in 2022) or 25 years and older (49.4% in 2022). Employment among mothers peaked at 37.3% in 2017–2018 but declined to 20.4% by 2022. Among husbands, the majority had a schooling background (over 94% across all years), with secondary education increasing to 35.2% and higher education reaching 20.6% in 2022. Healthcare decision‐making was increasingly joint: decisions made by both women and their partners rose from 48.6% in 2011 to 64.8% in 2022. Male‐headed households remained predominant (88.6% in 2022), although female‐headed households increased. Families with fewer than two children rose to 72.5% in 2022, and households with more than five members remained common (53.5% in 2022). Access to improved drinking water remained high at 86.3% in 2022, and improved toilet facilities increased from 47.9% in 2011 to 71.6% in 2022. Overall, the data indicate increases in education levels, access to improved sanitation, and joint healthcare decision‐making among reproductive‐aged women between 2011 and 2022.

**TABLE 1:** Sociodemographic characteristics of women by BDHS survey year (2011–2022).

Variables	BDHS survey years				*p*‐value
	2011	2014	2017–2018	2022	
Area					
Urban	1058 (22.83)	1206 (26.11)	1356 (26.85)	970 (26.89)	0.349
Rural	3577 (77.17)	3415 (73.89)	3695 (73.15)	2638 (73.11)	
Division					
Dhaka	1415 (30.52)	1633 (35.34)	1293 (25.60)	903 (25.01)	< 0.001
Barisal	255 (5.49)	268 (5.80)	288 (5.70)	216 (5.98)	
Chittagong	1077 (23.23)	1009 (21.83)	1071 (21.20)	776 (21.51)	
Khulna	448 (9.67)	370 (8.01)	464 (9.19)	374 (10.06)	
Mymensingh			431 (8.53)	324 (8.98)	
Rajshahi	616 (13.30)	463 (10.03)	587 (11.62)	377 (10.44)	
Rangpur	487 (10.50)	449 (9.72)	534 (10.57)	409 (11.33)	
Sylhet	338 (7.30)	428 (9.26)	383 (7.59)	230 (6.38)	
Religion					
Islam	4228 (91.22)	4237 (91.69)	4640 (91.85)	3346 (92.73)	0.775
Others	407 (8.78)	384 (8.31)	412 (8.15)	262 (7.27)	
Mother's age					
15–19	891 (19.22)	968 (20.96)	904 (17.90)	610 (16.90)	< 0.001
20–24	1745 (37.64)	1554 (33.63)	1779 (35.21)	1218 (33.74)	
25+	1999 (43.14)	2099 (45.41)	2368 (46.89)	1781 (49.36)	
Mother's education					
No education	809 (17.44)	654 (14.15)	318 (6.30)	178 (4.92)	< 0.001
Primary	1396 (30.12)	1293 (27.98)	1395 (27.62)	812 (22.51)	
Secondary	2083 (44.95)	2204 (47.70)	2475 (48.99)	1955 (54.18)	
Higher	347 (7.49)	470 (10.16)	863 (17.09)	664 (18.39)	
Currently working					
Yes	359 (7.74)	1093 (23.66)	1884 (37.31)	736 (20.39)	< 0.001
No	4276 (92.26)	3526 (76.34)	3167 (62.69)	2873 (79.61)	
Husband school type					
School	3164 (94.30)	3352 (95.25)	4087 (94.87)	2900 (95.46)	0.423
Madrasa	191 (5.70)	167 (4.75)	221 (5.13)	138 (4.54)	
Husband education level					
No education	1275 (27.53)	1101 (23.84)	680 (13.67)	540 (15.13)	< 0.001
Primary	1377 (29.74)	1386 (30.00)	1678 (33.73)	1038 (29.07)	
Secondary	1387 (29.96)	1467 (31.77)	1696 (34.09)	1256 (35.17)	
Higher	591 (12.77)	665 (14.40)	921 (18.51)	737 (20.64)	
Husband occupation					
Not working			33 (0.67)	73 (2.03)	< 0.001
Farming/agriculture	1246 (27.02)	1160 (25.20)	947 (19.04)	641 (17.94)	
Worker/jobholder	3233 (70.10)	3301 (71.75)	3977 (79.97)	2826 (79.09)	
Others	133 (2.88)	140 (3.05)	16 (0.31)	33 (0.93)	
Marital duration					
0–4	1579 (34.06)	1782 (38.55)	1817 (35.97)	1409 (39.06)	< 0.001
5–9	1483 (32.00)	1271 (27.50)	1501 (29.71)	983 (27.25)	
10–14	934 (20.14)	926 (20.04)	996 (19.71)	689 (19.08)	
15–19	433 (9.35)	434 (9.40)	549 (10.87)	387 (10.72)	
20–24	146 (3.15)	157 (3.39)	153 (3.03)	122 (3.39)	
25+	60 (1.29)	51 (1.11)	36 (0.71)	18 (0.51)	
Wanted pregnancy					
Then	3279 (70.75)	3421 (74.04)	3997 (79.12)	2906 (80.53)	< 0.001
Later	751 (16.21)	694 (15.03)	651 (12.88)	450 (12.46)	
No more	605 (13.04)	505 (10.93)	404 (8.0)	253 (7.00)	
Length of preceding birth					
< 24 months	352 (11.93)	300 (10.85)	301 (9.70)	208 (9.54)	< 0.001
24–59 months	1509 (51.14)	1222 (44.19)	1337 (43.06)	868 (39.87)	
60+ months	1090 (36.93)	1244 (44.97)	1466 (47.23)	1101 (50.59)	
Birth order					
1	1671 (36.06)	1844 (39.91)	1931 (38.22)	1421 (39.36)	< 0.001
2	1366 (29.48)	1392 (30.12)	1658 (32.82)	1195 (33.13)	
3+	1597 (34.46)	1385 (29.97)	1463 (28.96)	993 (27.51)	
Decision maker for healthcare					
Woman alone	450 (9.80)	512 (11.22)	370 (7.42)	275 (7.69)	< 0.001
Woman and partner	2234 (48.63)	2332 (51.09)	3262 (65.41)	2318 (64.79)	
Partner alone	1528 (33.27)	1374 (30.11)	1010 (20.25)	859 (24.01)	
Someone else	381 (8.30)	346 (7.58)	345 (6.92)	126 (3.51)	
Household head sex					
Male	4294 (92.65)	4227 (91.46)	4387 (86.84)	3196 (88.56)	< 0.001
Female	341 (7.35)	395 (8.54)	665 (13.16)	413 (11.44)	
Household head age					
15–24	234 (5.05)	248 (5.38)	249 (4.93)	181 (5.01)	< 0.001
25–29	675 (14.56)	654 (14.14)	687 (13.59)	405 (11.21)	
30–34	806 (17.39)	768 (16.62)	751 (14.87)	486 (13.48)	
35+	2920 (63.00)	2951 (63.86)	3365 (66.61)	2537 (70.30)	
Number of children					
Less than 2	3038 (65.54)	3236 (70.03)	3589 (71.04)	2616 (72.49)	< 0.001
More than 2	1597 (34.46)	1385 (29.97)	1463 (28.96)	993 (27.51)	
Number of household members					
Greater than 5	2376 (51.26)	2233 (48.32)	2472 (48.95)	1679 (53.47)	0.021
Less than or equal to 5	2259 (48.74)	2388 (51.68)	2579 (51.05)	1930 (46.53)	
Wealth index					
Poorest	1056 (22.78)	1001 (21.66)	1042 (20.62)	730 (20.24)	0.644
Poorer	919 (19.82)	875 (18.93)	1036 (20.51)	760 (21.05)	
Middle	917 (19.79)	882 (19.08)	969 (19.19)	764 (21.18)	
Richer	900 (19.42)	954 (20.64)	1018 (20.16)	708 (19.63)	
Richest	843 (18.18)	910 (19.69)	986 (19.53)	646 (17.91)	
Media access					
Yes	1793 (38.68)	1880 (40.68)	2114 (41.85)	1537 (42.59)	0.18
No	2842 (61.32)	2741 (59.32)	2937 (58.15)	2072 (57.41)	
Source of drinking water					
Improved	4023 (86.79)	4061 (87.89)	4293 (84.98)	3113 (86.26)	0.038
Unimproved	612 (13.21)	560 (12.11)	759 (15.02)	496 (13.74)	
Toilet facility					
Improved	2219 (47.86)	2904 (62.85)	2992 (59.23)	2585 (71.64)	< 0.001
Unimproved	2417 (52.14)	1717 (37.15)	2060 (40.77)	1023 (28.36)	
Total	4652 (100)	4627 (100)	5051 (100)	3609 (100)	

As shown in Table 2, the proportion of women receiving at least four antenatal care (ANC) visits increased from 25.4% in 2011 to 40.5% in 2022 (*p* < 0.001), although a slight decline was observed compared to 2017–2018 (47.0%). Facility‐based deliveries also rose markedly, from 28.3% in 2011 to 64.8% in 2022 (*p* < 0.001), reflecting an increase in facility‐based deliveries over the study period. Similarly, the use of skilled birth assistance improved from 31.2% in 2011 to 70.0% in 2022 (*p* < 0.001). The trend shows a significant and consistent increase in maternal healthcare coverage over the decade, although the slight decline in adequate ANC visits between 2017–2018 and 2022.

**TABLE 2:** Trends in maternal healthcare utilization over time (2011–2022).

Year	Antenatal care visits			Facility based delivery			Skilled birth attendant		
	Less than 4 *N* (%)	4 or above *N* (%)	*p*‐value	No health care facility *N* (%)	Health care facility *N* (%)	*p*‐value	No *N* (%)	Yes *N* (%)	*p*‐value
2011	3472 (74.59)	1180 (25.41)	**< 0.001**	3529 (71.75)	1536 (28.25)	<**0.001**	3383 (68.77)	1536 (31.23)	<**0.001**
2014	3179 (68.78)	1448 (31.22)		3953 (62.47)	2069 (37.53)		2825 (57.72)	2069 (42.28)	
2017–2018	2677 (52.99)	2374 (47.01)		2688 (50.35)	2650 (49.65)		2525 (47.30)	2813 (52.70)	
2022	2147 (59.49)	1462 (40.51)		1300 (35.22)	2391 (64.78)		1108 (30.03)	2582 (69.97)	
Total	11,460 (63.96)	6457 (36.04)		10,570 (56.12)	8265 (43.88)		9842 (52.23)	9001 (47.77)	

*Note:* Values in bold represent statistically significant associations at the 5% significance level (*p* < 0.05).

Using pooled BDHS data from 2011 to 2022, Figure 2 shows that Dhaka division consistently shows the highest coverage across all three indicators (ANC: 31.75%, FBD: 31.13%, and SBA: 30.53%), indicating better maternal healthcare access. In contrast, Sylhet and Barisal divisions report the lowest percentages for ANC (3.18% and 4.76%, respectively), FBD (3.47% and 4.51%), and SBA (3.44% and 5.05%), reflecting regional disparities.

**FIGURE 2:**
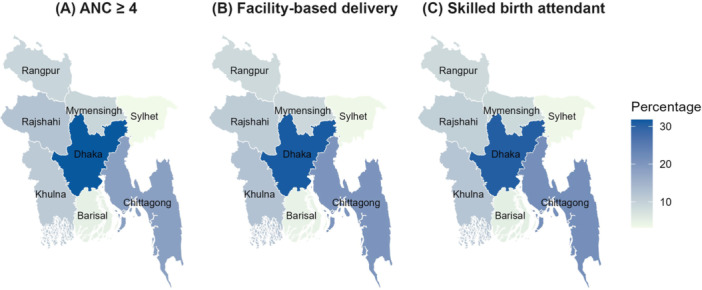
The divisional distribution of key maternal healthcare service utilization in Bangladesh: (A) at least four antenatal care (ANC) visits, (B) facility‐based delivery (FBD), and (C) skilled birth attendant (SBA).

Table 3 presents the proportion of women utilizing maternal healthcare services and the bivariate associations between service utilization, including antenatal care visits, facility‐based delivery, and skilled birth attendance and various demographic and socioeconomic characteristics. Overall, the likelihood of ANC visits (≥ 4) was higher among women living in urban (61.4%) compared to rural women (42.7%). Similarly, 68.9% of urban women delivered in health facilities vs. 44.1% of rural women, and skilled birth attendance was reported by 70.3% of urban women compared to 45.6% of rural women (*p* < 0.001 for all). Regional disparities were notable, with Dhaka showing the highest utilization rate of ANC (64.2%), facility‐based delivery (71.3%), and skilled attendance (72.1%). Utilization of services escalated with the education level of both women and their partners; for instances among women with higher education, 82.5% received professional birth attendants compared to 29.7% among those with no education. Skilled birth attendance was most common among women from the wealthiest households (80.4%), compared to just 27.8% among those from the poorest households (*p* < 0.001). Women with 1–2 children, those who had wanted pregnancies, and those with longer birth intervals (≥ 24 months) reported higher service use. Maternal healthcare utilization was more common in women who were not currently working, had shorter durations of marriage, had autonomy in decision‐making, and were regularly exposed to media. All these associations were significant (*p* < 0.05), underscoring the multifactorial nature of maternal health service access in Bangladesh.

**TABLE 3:** Distribution and association of maternal health care services and socio‐economic and demographic variables.

Variables	Antenatal care visits			Facility based delivery			Skilled birth attendant		
	< 4 *N* (%)	≥ 4 *N* (%)	*p*‐value	No *N* (%)	Yes *N* (%)	*p*‐value	No *N* (%)	Yes *N* (%)	*p*‐value
Area									
Urban	2223 (19.40)	2368 (36.68)	**< 0.001**	1865 (17.64)	2926 (35.40)	**< 0.001**	1652 (16.78)	3147 (34.96)	**< 0.001**
Rural	9237 (80.60)	4088 (63.32)		8706 (82.36)	5339 (64.60)		8190 (83.22)	5854 (65.04)	
Division									
Dhaka	3194 (27.87)	2050 (31.75)	< **0.001**	2953 (27.94)	2573 (31.13)	< **0.001**	2783 (28.27)	2748 (30.53)	< **0.001**
Barisal	719 (6.28)	307 (4.76)		693 (6.55)	373 (4.51)		611 (6.21)	455 (5.05)	
Chittagong	2744 (23.94)	1189 (18.41)		2498 (23.64)	1638 (20.30)		2251 (22.88)	1927 (21.41)	
Khulna	944 (8.24)	712 (11.03)		688 (6.51)	1027 (12.43)		631 (6.41)	1084 (12.04)	
Mymensingh	1232 (10.75)	603 (9.33)		1176 (11.13)	734 (8.88)		1133 (11.51)	778 (8.64)	
Rajshahi	1108 (9.67)	791 (12.25)		1077 (10.19)	887 (10.73)		1034 (10.51)	930 (10.33)	
Rangpur	1110 (9.68)	600 (9.29)		1106 (10.47)	707 (8.55)		1043 (10.60)	770 (8.56)	
Sylhet	408 (3.56)	205 (3.18)		378 (3.58)	287 (3.47)		356 (3.61)	309 (3.44)	
Religion									
Islam	10,607 (92.56)	5844 (90.51)	**< 0.004**	9878 (93.45)	7431 (89.91)	**< 0.001**	9191 (93.39)	8125 (90.27)	**< 0.001**
Others	852 (7.44)	613 (9.41)		692 (6.55)	834 (10.09)		651 (6.61)	876 (9.73)	
Mother's age									
15–19	2195 (19.15)	1179 (18.26)	0.453	1192 (18.85)	1527 (18.47)	0.636	1815 (18.44)	1703 (18.92)	0.319
20–24	4018 (35.06)	2277 (35.27)		3695 (34.95)	2956 (35.77)		3434 (34.89)	3223 (35.80)	
25+	5247 (45.79)	3001 (46.47)		4883 (46.20)	3782 (45.76)		4594 (46.67)	4076 (45.28)	
Mother's education									
No education	1692 (14.77)	266 (4.12)	< **0.001**	1735 (16.41)	379 (4.58)	< **0.001**	1693 (17.20)	422 (4.69)	
Primary	3711 (32.38)	1186 (18.36)		3691 (34.92)	1472 (17.81)		3530 (35.87)	1633 (18.14)	**< 0.001**
Secondary	5229 (45.63)	3489 (54.04)		4644 (43.94)	4474 (54.14)		4219 (42.87)	4906 (54.50)	
Higher	828 (7.23)	1516 (23.48)		500 (4.73)	1940 (23.47)		400 (4.06)	2040 (22.67)	
Currently working									
Yes	2533 (22.11)	1539 (23.83)	**0.042**	2539 (24.02)	1713 (20.73)	**< 0.001**	2394 (24.33)	1858 (20.64)	**< 0.001**
No	8924 (77.89)	4918 (76.17)		8030 (75.98)	6652 (79.27)		7446 (75.67)	7143 (79.36)	
Husband school type									
School	8028 (94.29)	5475 (95.95)	**< 0.001**	7136 (94.39)	6999 (95.44)	**0.018**	6550 (94.45)	7593 (95.32)	0.059
Madrasa	486 (5.71)	231 (4.05)		424 (5.61)	335 (4.56)		385 (5.55)	373 (4.68)	
Husband education level									
No education	2883 (25.33)	714 (11.12)	< **0.001**	2952 (28.11)	888 (10.81)	< **0.001**	2855 (29.20)	984 (11.00)	**< 0.001**
Primary	3937 (34.59)	1542 (24.04)		3923 (36.41)	1952 (23.78)		3596 (36.77)	2181 (30.86)	
Secondary	3460 (30.40)	2346 (36.57)		2969 (28.28)	3087 (37.59)		2690 (27.51)	3372 (37.72)	
Higher	1100 (9.67)	1813 (28.27)		757 (7.21)	2284 (27.82)		638 (6.52)	2403 (26.88)	
Husband occupation									
Not working	65 (0.57)	41 (0.64)	< **0.001**	28 (0.27)	79 (0.96)	< **0.001**	26 (0.27)	81 (0.91)	< **0.001**
Farming/agriculture	3044 (26.81)	950 (14.83)		3120 (29.77)	1133 (13.84)		2993 (30.68)	1259 (14.12)	
Worker/jobholder	8015 (70.60)	5321 (83.08)		7127 (68.02)	6847 (83.62)		6563 (67.25)	7419 (83.21)	
Others	229 (2.02)	93 (1.45)		204 (1.94)	129 (1.58)		176 (1.80)	157 (1.76)	
Marital duration									
0–4	3957 (34.53)	2603 (40.74)	< **0.001**	3418 (32.34)	3525 (42.65)	< **0.001**	3070 (31.19)	3872 (43.02)	< **0.001**
5–9	3366 (29.37)	1872 (28.99)		3115 (29.47)	2388 (28.89)		2936 (29.83)	2572 (28.58)	
10–14	2319 (20.24)	1224 (18.96)		2280 (21.57)	1459 (17.65)		2172 (22.07)	1568 (17.42)	
15–19	1259 (10.99)	545 (8.44)		1210 (11.44)	672 (8.13)		1141 (11.59)	743 (8.25)	
20–24	420 (3.66)	158 (2.45)		410 (3.97)	176 (2.13)		398 (4.04)	198 (2.19)	
25+	138 (1.21)	27 (0.42)		127 (1.21)	45 (0.55)		125 (1.27)	47 (0.53)	
Wanted pregnancy									
Then	8383 (73.16)	5220 (80.85)	< **0.001**	7659 (72.47)	6722 (81.33)	< **0.001**	7095 (72.10)	7289 (80.98)	< **0.001**
Later	1703 (14.87)	843 (13.05)		1570 (14.86)	1071 (12.96)		1465 (14.88)	1179 (13.10)	
No more	1372 (11.98)	394 (6.10)		1340 (12.68)	472 (5.71)		1281 (13.02)	533 (5.92)	
Length of preceding birth									
< 24 months	885 (11.85)	276 (7.08)	< **0.001**	873 (12.15)	339 (7.90)	< **0.001**	825 (12.13)	387 (8.27)	< **0.001**
24–59 months	3529 (47.23)	1408 (39.91)		3512 (48.85)	1697 (39.62)		3333 (48.97)	1880 (40.23)	
60+ months	3058 (40.92)	1844 (52.27)		2804 (39.00)	2247 (52.47)		2648 (38.91)	2406 (51.49)	
Birth order									
1	3964 (34.59)	2902 (44.95)	< **0.001**	3372 (31.90)	3939 (47.66)	< **0.001**	3030 (30.79)	4281 (47.56)	< **0.001**
2	3481 (30.38)	2131 (33.00)		3166 (29.95)	2670 (32.30)		2968 (30.16)	2870 (31.88)	
3+	4014 (35.03)	1424 (22.05)		4032 (38.15)	1657 (20.05)		3843 (39.05)	1850 (20.56)	
Decision maker for healthcare									
Woman alone	1015 (8.97)	592 (9.24)	< **0.001**	937 (8.97)	750 (9.15)	< **0.001**	863 (8.88)	1687 (9.23)	< **0.001**
Woman and partner	6205 (54.84)	3941 (61.51)		5694 (54.53)	4959 (60.51)		5285 (54.37)	5373 (60.21)	
Partner alone	3318 (29.32)	1453 (22.68)		3097 (29.65)	1948 (23.77)		2912 (29.96)	2134 (23.92)	
Someone else	777 (6.87)	421 (6.57)		715 (6.84)	538 (6.57)		661 (6.80)	592 (6.64)	
Household head sex									
Male	10,355 (90.36)	5749 (89.03)	**0.0254**	9569 (90.52)	7364 (89.10)	**0.013**	8925 (90.68)	8015 (89.05)	**0.004**
Female	1105 (9.64)	708 (10.97)		1002 (9.48)	901 (10.90)		917 (9.32)	986 (9.32)	
Household head age									
15–24	638 (5.57)	274 (4.25)	**0.004**	587 (5.55)	377 (4.56)	< **0.001**	549 (5.58)	415 (4.61)	< **0.001**
25–29	1583 (13.81)	836 (12.95)		1572 (14.87)	989 (11.96)		1468 (14.92)	1092 (12.13)	
30–34	1811 (15.80)	1001 (15.50)		1741 (16.48)	1228 (14.85)		1636 (16.62)	1337 (14.86)	
35+	7428 (64.82)	4345 (67.30)		6670 (63.10)	5672 (68.62)		6189 (62.88)	6157 (68.40)	
Number of children									
Less than 2	7445 (64.97)	5033 (77.95)	**< 0.001**	6383 (60.39)	6529 (79.00)	**< 0.001**	5855 (59.49)	7060 (79.44)	**< 0.001**
More than 2	4014 (35.03)	1424 (22.05)		4187 (39.61)	1736 (21.00)		3987 (40.51)	1941 (21.56)	
Number of household members									
Greater than 5	5717 (49.89)	3044 (47.14)	**0.004**	5405 (51.14)	3932 (47.57)	**< 0.001**	5032 (51.13)	4309 (47.87)	**0.001**
Less than or equal to 5	5743 (50.11)	3413 (52.86)		5165 (48.86)	4333 (52.43)		4810 (48.87)	4692 (52.13)	
Wealth index									
Poorest	3066 (26.76)	763 (11.81)	< **0.001**	3185 (30.13)	877 (10.61)	< **0.001**	3081 (31.30)	980 (10.89)	< **0.001**
Poorer	2674 (23.33)	915 (14.18)		2581 (24.41)	1229 (14.88)		2428 (24.67)	1383 (15.37)	
Middle	2380 (20.77)	1153 (17.85)		2136 (20.21)	1578 (19.09)		1968 (20.00)	1746 (19.40)	
Richer	2044 (17.84)	1536 (23.79)		1729 (16.36)	2000 (24.19)		1562 (15.87)	2166 (24.07)	
Richest	1296 (11.31)	2090 (32.36)		939 (8.89)	2581 (31.23)		903 (8.16)	2725 (30.27)	
Media access									
Yes	3768 (32.88)	3556 (55.08)	**< 0.001**	3201 (30.28)	4421 (46.51)	**< 0.001**	2890 (29.36)	4738 (47.36)	**< 0.001**
No	7692 (67.18)	2900 (44.92)		7270 (69.72)	3844 (53.49)		6952 (70.64)	4263 (52.64)	
Source of drinking water									
Improved	9980 (87.09)	5510 (85.34)	**0.021**	9242 (87.43)	7027 (85.02)	**0.001**	8589 (87.27)	7688 (85.41)	**0.013**
Unimproved	1480 (12.91)	947 (14.66)		1329 (12.57)	1238 (14.98)		1253 (12.73)	1313 (14.59)	
Toilet facility									
Improved	6257 (54.60)	4443 (68.81)	**< 0.001**	5398 (51.07)	5789 (70.05)	**< 0.001**	4905 (48.83)	6288 (69.86)	**< 0.001**
Unimproved	5202 (45.40)	2014 (31.19)		5172 (48.93)	2476 (29.95)		4937 (50.17)	2713 (30.14)	
Total	11,460 (63.96)	6457 (36.04)		10,570 (56.12)	8265 (43.88)		9842 (52.23)	9001 (47.77)	

*Note:* Values in bold represent statistically significant associations at the 5% significance level (*p* < 0.05).

Table 4 shows AORs for maternal health care access by sociodemographic, household factors, and survey years in Bangladesh. Compared to 2011, women in 2022 were significantly more likely to receive four or more antenatal care (ANC) visits (AOR: 1.57; 95% CI: 1.29–1.90), facility‐based delivery (AOR: 5.36; 95% CI: 4.34–6.63), and receive skilled birth attendance (AOR: 5.61; 95% CI: 4.63–6.79). Although the likelihood of at least four or more ANC visits declined slightly in 2022 compared to 2017 (AOR: 2.31; 95% CI: 1.92–2.78), facility‐based delivery and skilled birth attendance continued rising steadily. Urban women had higher odds of ANC visits (AOR: 1.35; 95% CI: 1.16–1.58), facility delivery (AOR: 1.28; 95% CI: 1.10–1.51), and skilled attendance (AOR: 1.34; 95% CI: 1.16–1.55) than rural women. Regionally, women in Chittagong were less likely to utilize ANC (AOR: 0.74; 95% CI: 0.59–0.91) and facility delivery (AOR: 0.79; 95% CI: 0.64–0.97) than those in Dhaka, while women in Khulna were more likely to deliver at facilities (AOR: 1.82; 95% CI: 1.43–2.31) and have skilled birth assistance (AOR: 1.88; 95% CI: 1.51–2.34). Muslim women had lower odds of ANC (AOR: 0.78; 95% CI: 0.63–0.97), facility delivery (AOR: 0.55; 95% CI: 0.43–0.71), and skilled attendance (AOR: 0.64; 95% CI: 0.50–0.81) compared to non‐Muslims. Maternal education was a strong predictor: women who had higher education had markedly higher probability of adequate ANC visits (AOR: 4.59; 95% CI: 3.27–6.43), facility delivery (AOR: 4.44; 95% CI: 3.14–6.29), and skilled birth attendance (AOR: 6.07; 95% CI: 4.52–8.16) vs. those with no education. Women with a preceding birth interval of 60+ months had higher likelihood of necessary ANC visits (AOR: 1.58; 95% CI: 1.24–2.02), facility‐based delivery (AOR: 1.59; 95% CI: 1.27–2.01), and skilled attendance (AOR: 1.47; 95% CI: 1.20–1.81) than those with intervals under 24 months. Women whose partners alone decided on healthcare had lower odds of skilled attendance (AOR: 0.80; 95% CI: 0.65–0.99) than women deciding alone. Employment status showed no association with ANC but who were employed had lower odds of facility‐based delivery (AOR: 0.76; 95% CI: 0.65–0.88) and skilled attendance (AOR: 0.74; 95% CI: 0.64–0.86). Husband's education was positively linked to ANC utilization (AOR: 1.45; 95% CI: 1.12–1.88) compared to madrasa education. Husbands in jobs or business were associated with higher ANC uptake, while those in farming/agriculture showed lower odds of facility delivery and skilled attendance. Larger households (> 5 members) had lower likelihood of facility delivery (AOR: 0.77; 95% CI: 0.66–0.89) and skilled birth attendance (AOR: 0.76; 95% CI: 0.67–0.86). Women with more than two children showed decreased likelihood of facility‐based delivery (AOR: 0.77; 95% CI: 0.66–0.91) and skilled birth attendance (AOR: 0.78; 95% CI: 0.68–0.90). A clear wealth gradient was evident, with richest households having higher odds of ANC (AOR: 2.59; 95% CI: 1.99–3.38), facility delivery (AOR: 4.64; 95% CI: 3.53–6.10), and skilled attendance (AOR: 4.34; 95% CI: 3.40–5.43) than poorest. Exposure to media positively influenced ANC (AOR: 1.33; 95% CI: 1.15–1.54), facility delivery (AOR: 1.26; 95% CI: 1.08–1.46), and skilled attendance (AOR: 1.24; 95% CI: 1.07–1.42). Improved sanitation was associated with skilled attendance (AOR: 1.15; 95% CI: 1.01–1.31), while improved drinking water was related to better ANC utilization (AOR: 1.43; 95% CI: 1.11–1.84).

**TABLE 4:** Multivariable logistic regression‐adjusted estimates of factors associated with maternal health care access.

Variables	Antenatal care visits		Facility based delivery		Skilled birth attendant	
	AOR (95% CI)	*p*‐value	AOR (95% CI)	*p*‐value	AOR (95% CI)	*p*‐value
Year						
2011	Reference		Reference		Reference	
2014	1.30 (1.05–1.62)	**0.017**	1.77 (1.44–2.18)	**< 0.001**	1.79 (1.50–2.16)	**< 0.001**
2017–2018	2.31 (1.92–2.78)	**< 0.001**	2.70 (2.21–3.29)	**< 0.001**	2.64 (2.20–3.17)	**< 0.001**
2022	1.57 (1.29–1.90)	**< 0.001**	5.36 (4.34–6.63)	**< 0.001**	5.61 (4.63–6.79)	**< 0.001**
Area						
Urban	1.35 (1.16–1.58)	**< 0.001**	1.28 (1.09–1.51)	**0.002**	1.34 (1.16–1.55)	**< 0.001**
Rural	Reference		Reference		Reference	
Division						
Dhaka	Reference		Reference		Reference	
Barisal	0.84 (0.66–1.09)	0.213	0.79 (0.61–1.04)	0.092	0.99 (0.78–1.26)	0.972
Chittagong	0.74 (0.59–0.91)	**0.005**	0.79 (0.64–0.97)	**0.027**	0.91 (0.74–1.10)	0.321
Khulna	1.12 (0.89–1.42)	0.336	1.82 (1.43–2.31)	**< 0.001**	1.88 (1.51–2.35)	**< 0.001**
Mymensingh	1.12 (0.88–1.44)	0.349	1.01 (0.79–1.31)	0.910	0.96 (0.77–1.20)	0.719
Rajshahi	1.27 (0.98–1.65)	0.075	1.01 (0.79–1.31)	0.913	1.05 (0.83–1.32)	0.705
Rangpur	1.19 (0.93–1.51)	0.169	1.03 (0.81–1.30)	0.796	1.04 (0.83–1.29)	0.744
Sylhet	1.01 (0.74–1.37)	0.952	0.79 (0.59–1.07)	0.132	0.78 (0.59–1.04)	0.089
Religion						
Islam	0.78 (0.63–0.97)	**0.027**	0.55 (0.43–0.71)	**< 0.001**	0.64 (0.50–0.81)	**< 0.001**
Others	Reference		Reference		Reference	
Education						
No education	Reference		Reference		Reference	
Primary	1.54 (1.16–2.05)	**0.003**	1.16 (0.88–1.54)	0.296	1.38 (1.11–1.71)	**0.003**
Secondary	2.42 (1.80–3.23)	**< 0.001**	1.79 (1.35–2.39)	**< 0.001**	2.25 (1.83–2.77)	**< 0.001**
Higher	4.59 (3.27–6.43)	**< 0.001**	4.44 (3.14–6.29)	**< 0.001**	6.07 (4.52–8.16)	**< 0.001**
Currently working						
Yes	1.03 (0.89–1.19)	0.712	0.76 (0.65–0.88)	**< 0.001**	0.74 (0.64–0.86)	**< 0.001**
No	Reference		Reference		Reference	
Husband school type						
School	1.45 (1.12–1.88)	**0.006**	1.19 (0.88–1.62)	0.251		
Madrasa	Reference		Reference			
Husband occupation						
Not working	Reference		Reference		Reference	
Farming/agriculture	1.58 (0.82–3.01)	0.169	0.38 (0.19–0.78)	**0.008**	0.54 (0.29–0.98)	**0.044**
Worker/jobholder	2.07 (1.09–3.90)	**0.001**	0.54 (0.27–1.08)	0.084	0.77 (0.43–1.39)	0.391
Others	1.65 (0.72–3.76)	0.233	0.54 (0.23–1.25)	0.148	0.94 (0.46–1.92)	0.873
Marital duration						
0–4	Reference		Reference		Reference	
5–9	0.88 (0.70–1.11)	0.286	0.99 (0.78–1.26)	0.936	0.97 (0.78–1.21)	0.819
10–14	0.95 (0.73–1.23)	0.686	1.05 (0.79–1.38)	0.755	0.96 (0.75–1.24)	0.766
15–19	0.95 (0.69–1.30)	0.743	0.94 (0.67–1.32)	0.735	1.01 (0.76–1.36)	0.908
20–24	1.03 (0.68–1.57)	0.871	0.89 (0.59–1.34)	0.579	0.95 (0.65–1.39)	0.808
25+	0.93 (0.47–1.84)	0.826	0.90 (0.43–1.90)	0.786	1.16 (0.69–1.96)	0.578
Wanted pregnancy						
Later	0.899 (0.76–1.06)	0.211	0.94 (0.79–1.10)	0.430	1.02 (0.87–1.19)	0.812
No more	0.79 (0.62–1.01)	0.058	0.99 (0.78–1.28)	0.991	0.93 (0.77–1.13)	0.471
Then	Reference		Reference		Reference	
Length of preceding birth						
Less than 24 months	Reference		Reference		Reference	
24–59 months	1.29 (1.004–1.65)	**0.046**	1.22 (0.95–1.56)	0.120	1.14 (0.93–1.39)	0.219
60+ months	1.58 (1.24–2.02)	**< 0.001**	1.59 (1.27–2.01)	**< 0.001**	1.47 (1.20–1.81)	**< 0.001**
Decision maker for healthcare						
Woman alone	Reference		Reference		Reference	
Woman and partner	1.12 (0.92–1.37)	0.263	0.94 (0.77–1.15)	0.547	0.92 (0.76–1.11)	0.382
Partner alone	0.96 (0.77–1.20)	0.745	0.83 (0.66–1.04)	0.109	0.80 (0.65–0.99)	**0.037**
Someone else	0.93 (0.67–1.30)	0.677	0.75 (0.54–1.04)	0.087	0.79 (0.58–1.08)	0.137
Household head sex						
Male	0.88 (0.72–1.07)	0.174	0.93 (0.76–1.13)	0.458	0.93 (0.77–1.12)	0.425
Female	Reference		Reference		Reference	
Household head age						
15–24	Reference		Reference		Reference	
25–29	1.25 (0.84–1.88)	0.268	1.02 (0.69–1.50)	0.916	0.94 (0.66–1.34)	0.714
30–34	1.22 (0.82–1.80)	0.315	1.01 (0.69–1.47)	0.975	0.99 (0.70–1.41)	0.977
35+	1.21 (0.82–1.79)	0.327	1.24 (0.85–1.82)	0.262	1.20 (0.85–1.70)	0.299
Number of children						
More than 2	0.95 (0.82–1.09)	0.471	0.77 (0.66–0.91)	**0.002**	0.78 (0.68–0.90)	**0.001**
Less than 2	Reference		Reference		Reference	
Number of household members						
Greater than 5	0.86 (0.74–1.0)	0.052	0.77 (0.66–0.89)	**0.001**	0.76 (0.67–0.86)	**< 0.001**
Less than or equal to 5	Reference		Reference		Reference	
Wealth index						
Poorest	Reference		Reference		Reference	
Poorer	0.96 (0.78–1.18)	0.735	1.20 (0.96–1.51)	0.113	1.31 (1.10–1.56)	**0.002**
Middle	1.23 (0.99–1.53)	0.058	1.79 (1.41–2.30)	**< 0.001**	1.91 (1.56–2.33)	**< 0.001**
Richer	1.48 (1.19–1.85)	**< 0.001**	2.71 (2.15–3.39)	**< 0.001**	2.51 (2.06–3.07)	**< 0.001**
Richest	2.59 (1.99–3.38)	**< 0.001**	4.64 (3.53–6.10)	**< 0.001**	4.34 (3.40–5.43)	**< 0.001**
Media						
Yes	1.33 (1.15–1.54)	**< 0.001**	1.26 (1.08–1.46)	**0.002**	1.24 (1.07–1.42)	**0.003**
No	Reference		Reference		Reference	
Source of drinking water						
Improved	1.43 (1.11–1.84)	**0.006**	0.88 (0.67–1.17)	0.385	0.91 (0.72–1.16)	0.463
Unimproved	Reference		Reference		Reference	
Toilet facility						
Improved	1.05 (0.89–1.24)	0.550	1.08 (0.92–1.27)	0.351	1.15 (1.01–1.31)	**0.04**
Unimproved	Reference		Reference		Reference	

*Note:* Values in bold represent statistically significant associations at the 5% significance level (*p* < 0.05).

**FIGURE 3:**
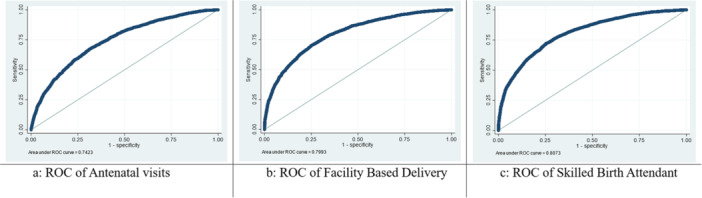
ROC curves for maternal healthcare service utilization indicators. (a) ROC of Antenatal visits. (b) ROC of Facility Based Delivery. (c) ROC of Skilled Birth Attendant.

Table 5 shows that model validity tests provided strong support for the reliability of the findings. As shown in Figure 3, the models demonstrated good fit and strong predictive power, with AUROC values of 0.7423 for ANC visits, 0.7993 for FBD, and 0.8073 for SBA, all indicating strong model performance. The classification accuracy ranged from 70.07% for ANC visits, 72.80% for facility‐based delivery and 73.69% skilled birth assistance, demonstrating the models' ability to accurately predict maternal healthcare utilization. The Hosmer–Lemeshow tests confirmed the goodness of fit for each model, with *p*‐values indicating that the models are well‐calibrated. Notably, two variables‐birth order and partner's education‐were excluded from the final multivariable models due to multicollinearity, as indicated by VIF diagnostics. Their removal helped ensure model stability and interpretability.

**TABLE 5:** Assessment of model performance.

Maternal healthcare service outcome models	AIC	BIC	AUROC	Hosmer–Lemeshow goodness of fit test	Classification accuracy
Antenatal care visits	9377.62	9699.55	0.74 (0.73–0.75)	Chi‐squared = 4.49 *p*‐value = 0.811	70.07%
Facility‐based delivery	9102.47	9426.01	0.80 (0.79–0.81)	Chi‐squared = 5.25 *p*‐value = 0.731	72.80%
Skilled birth attendant	11,758.69	12,088.04	0.81 (0.80–0.82)	Chi‐squared = 10.52 *p*‐value = 0.230	73.69%

## 4 Discussion

According to our findings, maternal healthcare facilities utilization in Bangladesh from 2011 to 2022 showed significant improvement, as well as disparities influenced by socioeconomic and geographic factors. Compared to 2011, the prevalence of maternal healthcare utilization increased significantly. Although there was a slight decline in the utilization of ANC visits in 2022 compared to 2017, the decline in adequate antenatal care observed in 2022 relative to 2017–2018 may reflect disruptions related to the COVID‐19 pandemic, including mobility restrictions and concerns about infection, as reported in earlier studies [34, 35]. Overall, these temporal patterns are consistent with prior national evidence and suggest continued progress toward maternal health–related sustainable development goals (SDGs) [19, 21].

Despite these gains, marked disparities remain. Women residing in urban areas were more likely to utilize antenatal care, facility‐based delivery and skilled birth attendance than their rural counterparts, consistent with earlier studies documenting persistent urban‐rural gaps in access to healthcare infrastructure and services [20, 36, 37]. Moreover, regional inequalities are also present with woman living in Khulna showing higher odds of facility‐based delivery and skilled birth assistance, while woman in the Chittagong division had the lower odds of attending at least four ANC visits compared to those in Dhaka. These findings are consistent with earlier evidence highlighting regional disparities in maternal healthcare utilization [38]. Religious affiliation was associated with differences in utilization; however, these patterns may reflect broader sociocultural norms rather than religious belief per se [36, 39, 40].

Socioeconomic position and education emerged as some of the strongest correlates of maternal healthcare utilization. Higher maternal education was consistently associated with greater use of all three services, reinforcing evidence that education enhances health awareness, care‐seeking behavior, and navigation of health systems [10, 41, 42]. Similarly, women from the wealthiest households had higher likelihood to have access to these services than those from the poorest households. These results align with previous studies that emphasize the association of wealth disparities on access to maternal healthcare services [13, 27].

Though employment did not significantly affect ANC utilization but woman who were working preceding the survey had 24% less SBA and 26% less institutional deliveries compared to woman who were not. Previous study also shows that women who were working were less likely to use institutional delivery services [43]. Husband's education and occupation can also have a notable influence on maternal healthcare utilization across different service indicators [27, 42, 44]. In addition, longer birth intervals particularly those exceeding 5 years were associated with higher utilization, consistent with evidence showing maternal and child health outcomes associated modern postpartum family planning methods, which may facilitate longer birth spacing [45].

Households where the mother makes the decision for healthcare showed higher likelihood of having skilled birth assistance than households with husband alone making decisions. Prior studies also indicate that the women's ability to make decisions is favorably associated with maternal healthcare utilization [46, 47]. Both the number of children and the number of household members have been shown to influence maternal healthcare utilization. Past investigations also show that women with less number of children and women belonging from nuclear family had a higher tendency of using maternal health care facilities [48, 49]. Finally, access to mass media and improved household amenities showed positive associations with maternal healthcare utilization. Media exposure may facilitate awareness of available services and encourage timely care‐seeking [33, 50]. While improved water and sanitation conditions likely reflect broader household socioeconomic status and health‐supportive environments as found in our study [51].

### 4.1 Strengths and Limitations

This study offers a thorough understanding of patterns over time by utilizing data from several BDHS surveys conducted between 2011 and 2022. The nationally representative sample of women enhances the generalizability of the findings. Multivariable logistic regression model allowed the study to find the determinants associated with usage of maternal healthcare services. The identifications of key social and economic, as well as demographic factors of access to maternal healthcare services can help policy and intervention strategies. The model's performance (validity, goodness of fit) confirms the reliability of the findings.

Although studying has several strengths, it also has some limitations. Its cross‐sectional design limits causal inference, and self‐reported data may introduce recall bias. Unobserved community‐level factors and contextual variables, such as quality of care, women's autonomy, and health system characteristics, were not captured. Variations in BDHS survey design may affect trend and comparability. Exclusion of birth order and partner's education due to multicollinearity could omit relevant predictors. Finally, the findings are specific to Bangladesh and may not generalize to other low‐ and middle‐income countries.

### 4.2 Recommendation

The evidence underscores the need to improve accessibility and utilization of maternal healthcare in Bangladesh. Efforts should focus on enhancing women's education, targeting rural and underserved areas like Chittagong, and raising awareness among economically disadvantaged and less educated women. Workforce‐related barriers can be addressed through maternity‐friendly policies and accessible resources, while women's voices should be included in decision‐making. Strengthening media outreach, public health communication, sanitation, and healthcare infrastructure will create supportive environments, and culturally sensitive strategies can engage communities with lower service uptake, ensuring equitable maternal healthcare for all.

## 5 Conclusion

This study shows substantial improvements in maternal healthcare access in Bangladesh during the previous decade particularly in skilled birth attendance, facility‐based delivery, and antenatal care. Considerable disparities are still present across geographic, socioeconomic and demographic characteristics, including maternal and partner education, affluence, urban residency, media exposure, and birth spacing were positively correlated with increased service utilization, whereas rural residency, lower educational attainment, and religion disparities constituted obstacles. Despite expanded service availability, the recent decline in ANC visits highlights the need for targeted policies. Strengthening education, improving access for underserved groups, and addressing regional and cultural disparities are essential for equitable and sustainable maternal health gains.

## Author contributions

**Kamrul Hassan Prantik:** conceptualization, methodology, software, data curation, investigation, formal analysis, visualization, project administration, resources, writing – original draft, writing – review and editing. **Anika Tabassum Nabi:** formal analysis, investigation, methodology, Writing – original draft, Writing – review and editing. **Golam Morshed Suhel** and **Mahmud Afroz:** methodology, resources, writing – review and editing, visualization. **Mohammad Nayeem Hasan:** conceptualization, formal analysis, supervision, writing – review and editing, resources, visualization, investigation, methodology, validation.

## Funding

The authors received no specific funding for this work.

## Ethics Statement

Ethical approval was not required for this study as it utilized publicly available secondary data from the Demographic and Health Surveys (DHS) program.

## Conflicts of Interest

The authors declare no conflicts of interest.

## Transparency Statement

The lead author Mohammad Nayeem Hasan affirms that this manuscript is an honest, accurate, and transparent account of the study being reported; that no important aspects of the study have been omitted; and that any discrepancies from the study as planned (and, if relevant, registered) have been explained.

## Supporting information

Supplementary file.

## Data Availability

This study utilized data from the Bangladesh Demographic and Health Surveys (BDHS) conducted in 2011, 2014, 2017–2018, and 2022. These nationally representative datasets are publicly available through the DHS Program https://www.dhsprogram.com/data/available-datasets.cfm.
